# Naucline, a New Indole Alkaloid from the Bark of *Nauclea officinalis* ^†^

**DOI:** 10.3390/molecules17044028

**Published:** 2012-04-02

**Authors:** Sook Yee Liew, Mat Ropi Mukhtar, A. Hamid A. Hadi, Khalijah Awang, Mohd Rais Mustafa, Kazumasa Zaima, Hiroshi Morita, Marc Litaudon

**Affiliations:** 1 Department of Chemistry, Faculty of Science, University of Malaya, 50603 Kuala Lumpur, Malaysia; Email: joeyliew5382@um.edu.my (S.Y.L.); ahamid@um.edu.my (A.H.A.H.); 2 Department of Pharmacology, Faculty of Medicine, University of Malaya, 50603 Kuala Lumpur, Malaysia; Email: rais@um.edu.my; 3 Faculty of Pharmaceutical Sciences, Hoshi University, Shinagawa-ku, Tokyo 142-8501, Japan; Email: moritah@hoshi.ac.jp; 4 Institut de Chimie de la Substances Naturelles, Centre Nationale de la Recherches Scientifique, 91198, Gif-sur Yvette, Cedex, France; Email: marc.litaudon@icsn.cnrs-gif.fr

**Keywords:** naucline, angustine, angustidine, nauclefine, naucletine, Rubiaceae, vasorelaxant activity

## Abstract

A new indole alkaloid, naucline (**1**) together with four known alkaloids, angustine (**2**), angustidine (**3**), nauclefine (**4**) and naucletine (**5**), were isolated from the bark of *Nauclea officinalis*. The structures of all isolated compounds were elucidated with various spectroscopic methods such as 1D- and 2D- NMR, IR, UV and LCMS-IT-TOF. In addition to that of alkaloid **1**, the complete ^13^C-NMR data of naucletine (**5**) were also reported. Naucline (**1**) showed a moderate vasorelaxant activity (90% relaxation at 1 × 10^−5^ M) whereas, angustine (**2**), nauclefine (**4**), and naucletine (**5**) showed potent vasorelaxant activity (more than 90% relaxation at 1 × 10^−5^ M) on an isolated rat aorta.

## 1. Introduction

The Rubiaceae family is known as Madder or Bedstraw, comprising 650 genera and 10,500 species worldwide [1]. Most of them are distributed primarily in the tropical regions and are mainly woody trees and shrubs [2]. A number of monoterpenoid indole alkaloids have been isolated from the *Nauclea* genus [3]. Some of these alkaloids were reported to exhibit certain biological activities such as anticonvulsant, antiproliferative, antimalarial, antimicrobial and antiparasitic properties [4,5,6,7,8]. *Nauclea officinalis*, a traditional Chinese medicine, is reported to contain alkaloids and terpenoids as major components [9,10].

**Figure 1:**
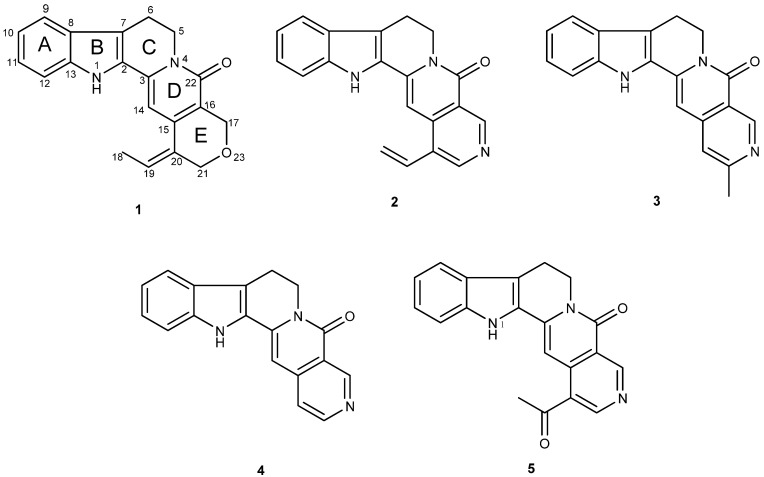
Structures of naucline (**1**), angustine (**2**), angustidine (**3**), nauclefine (**4**), and naucletine (**5**).

In our continuous effort of searching for interesting chemical constituents of the Rubiaceae family from Malaysia [11], a new monoterpenoid indole alkaloid has been isolated from the bark of *Nauclea officinalis*. In addition to the new compound, four known alkaloids, angustine (**2**) [12,13,14,15,16,17,18], angustidine (**3**) [13,17,19], nauclefine (**4**) [15,19,20,21] and naucletine (**5**) [15,19,22] were also isolated (Figure 1). In the present paper, we report the isolation and characterization of this new indoloquinolizidinone alkaloid, namely naucline (**1**) and the vasorelaxant activity of the indole alkaloids **1**, **2**, **4** and **5**. The ^13^C-NMR data of naucletine (**5**) are also presented.

## 2. Results and Discussion

Naucline (**1**) was isolated as a brownish amorphous solid. The LCMS-IT-TOF spectrum revealed a pseudomolecular ion peak [M+H]^+^ at *m/z* 319.1450, corresponding to the molecular formula of C_20_H_18_N_2_O_2_. In the IR spectrum, an absorption band due to a conjugated carbonyl stretching vibration was observed at 1638 cm^−1^.

In the ^1^H-NMR spectrum, the presence of four aromatic protons, a broad peak of -NH- and one -CH_2_-CH_2_-N- group were observed, suggesting a *β*-carboline skeleton [10]. Two of the four aromatic protons in ring A appeared as doublets at δ_H_ 7.48 and 7.33, and the other as two doublet of doublets (dd) at δ_H_ 7.10 and 7.21 were attributed to H-9, H-12, H-10, and H-11, respectively. H-14 of ring D was revealed as a singlet at δ_H_6.32 indicating that a double bond could be formed between C-15 and C-16. In addition, two upfield signals of H-19 (δ_H_ 5.76, q, *J* = 6.6 Hz) and methyl protons (δ_H_ 1.46, d, *J* = 6.6 Hz) characteristic of trisubstituted olefin group were observed [10]. The ^13^C-NMR and DEPT spectra of naucline (**1**) indicated a total of 20 carbon signals; one methyl, one carbonyl, four methylenes, six methines and eight quaternary carbons. The presence of a carbonyl carbon was observed at δ_C_ 163.1. The signals at δ_C_ 58.9 and 66.6 could be assigned as the resonances of two oxymethylenes, C-17 and C-21, respectively.

**Figure 2:**
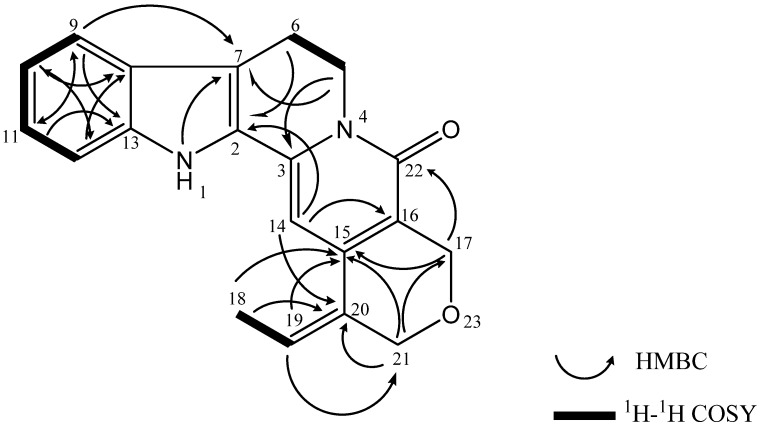
Selected 2D NMR correlations for naucline (**1**).

**Figure 3:**
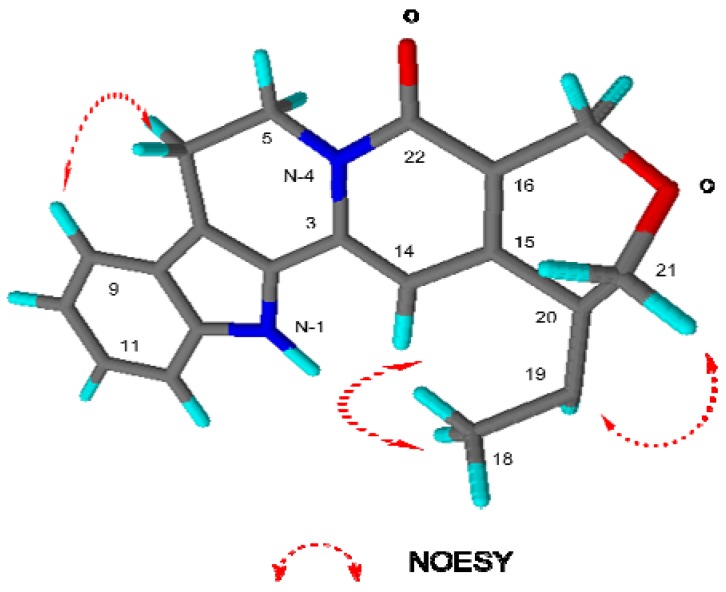
Selected NOESY correlations of naucline (**1**).

Selected 2D NMR correlations (COSY and HMBC) for naucline (**1**) are shown in Figure 2. Complete ^1^H- and ^13^C-NMR (Table 1) spectral assignment of **1** was accomplished through analysis of COSY, HMQC, HMBC and NOESY data. The presence of a trisubstituted olefin with a methyl group at C-19 (δ_C_ 119.5) and an oxymethylene at C-20 were determined by using HMBC correlations from H-14, H-18 (δ_H_ 1.46), and H-21 (δ_H_ 4.18, d) to C-20 (δ_C_ 148.0), and from H-18, H-19 (δ_H_ 5.76) and H-21 to C-15 (δ_C_ 136.6) (Figure 2). The COSY spectrum showed correlation peaks between H_2_-5/H_2_-6 and H_3_-18/H-19 respectively. NOESY correlations of naucline (**1**) is shown in Figure 3.

Four known alkaloids, angustine (**2**), angustidine (**3**), nauclefine (**4**), naucletine (**5**) were isolated as brownish amorphous solids. The LCMS-IT-TOF spectra showed molecular ion peaks, [M+H]^+^ at *m/z* 314.1316 [C_20_H_15_N_3_O], 302.1317 [C_19_H_15_N_3_O], 288.1155 [C_18_H_13_N_3_O], and 330.1309 [C_20_H_15_N_3_O_2_] respectively. The spectroscopic data of 5 were reported in comprehensive reviews [15,23], but its ^13^C-NMR data were lacking. In view of that, complete assignments were established through various NMR measurements; DEPT, HSQC, and HMBC spectra. The ^13^C-NMR spectra of naucletine (**5**) indicated the presence of 20 carbons. Two carbonyl carbons were observed at δ_C_ 199.6 (-CCOCH_3_) and 161.6 (-NCOC-), respectively.

**Table 1:** ^1^H-NMR (400 MHz) and ^13^C-NMR (100 MHz) Spectral Data of Naucline (**1**) and Naucletine (**5**) in CDCl_3_.

Position	1		5	
	^1^H (δ_H_, Hz)	^13^C (δ_C_)	^1^H (δ_H_, DMSO, Hz) ^a^	^13^C (δ_C_)
NH-1	9.72 (s)	-		
2	-	127.4	-	127.4
3	-	137.9	-	140.8
5a	4.03 (m)	40.7	4.39 (t, 6.9)	40.7
5b	4.59 (m)			
6	2.96 (m)	19.5	3.12 (t, 6.9)	19.8
7	-	114.4	-	116.9
8	-	125.6	-	125.7
9	7.48 (d, 7.8)	119.5	7.65 (d, 8.0)	119.3
10	7.10 (dd, 7.8, 7.3)	120.5	7.07 (m)	119.9
11	7.21 (dd, 7.3, 8.2)	124.7	7.23 (m)	120.9
12	7.33 (d, 8.2)	111.9	7.45 (d, 8.1)	112.0
13	-	138.5	-	139.0
14	6.32 (s)	102.0	7.73 (s)	95.6
15	-	136.6	-	141.1
16	-	125.7	-	117.1
17	4.36 (br d, 12.1)	58.9	9.21 (s)	154.0
	4.65 (br d, 12.1)			
18	1.46 (d, 6.6)	14.6	2.71 (s)	29.3
19	5.76 (q, 6.6)	125.9	-	199.6
20	-	148.0	-	138.8
21	4.18 (br d, 11.9)	66.6	9.41 (s)	155.4
	4.20 (br d, 11.9)			
22	-	163.1	-	161.6

^a^ ^1^H-NMR data are reported from Lavilla *et al.*

**Figure 4:**
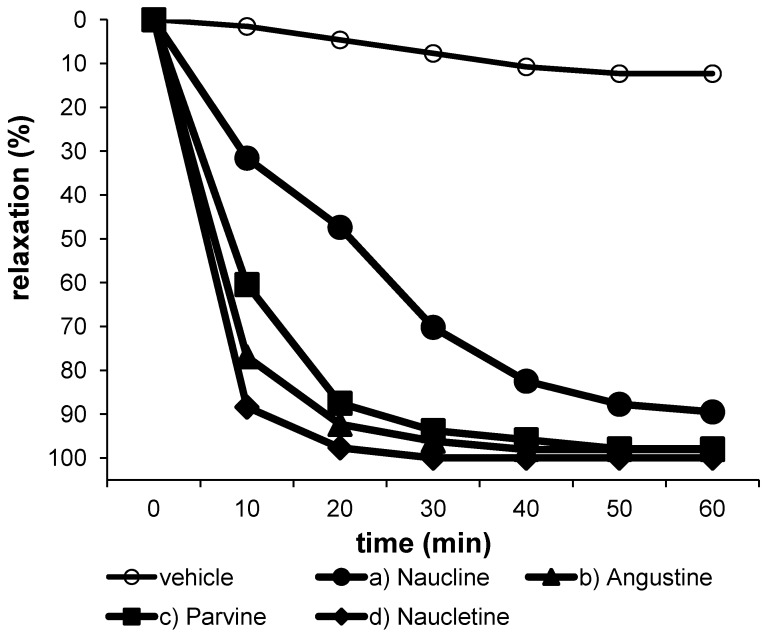
Relaxation responses induced by naucline (**1**; 1 × 10^−5^ M), angustine (**2**; 1 × 10^−5^ M), nauclefine (parvine) (**4**; 1 × 10^−5^ M), and naucletine (**5**; 1 × 10^−5^ M) in aortic rings precontracted with 3 × 10^−4^ M phenylephrine (PE).

Vasodilators are useful for treatment of cerebral vasospasm and hypertension, and for improvement of peripheral circulation [24]. After phenylephrine (PE) 3 × 10^−7^ M was applied to thoracic aortic rings with endothelium and after achieving a maximal response, we added naucline (**1**; 1 × 10^−5^ M), angustine (**2**; 1 × 10^−5^ M), nauclefine (**4**; 1 × 10^−5^ M), and naucletine (**5**; 1 × 10^−5^ M). Excellent activity could be observed for angustine (**2**), nauclefine (**4**), and naucletine (**5**) at the early stage within 10–;30 min after injection of each sample (more than 90% relaxation at 1 × 10^−5^ M), whereas naucline (**1**) showed a moderate vasorelaxant activity (90% relaxation at 1 × 10^−5^ M) (Figure 4). Vasodilation by these indole alkaloids seems to be influenced by substitution of a nitrogen atom in ring E. In the previous paper, we have reported vasorelaxant activities of some bisbenzylisoquinoline alkaloids such as α′-oxoperakensimines A–;C from *Alseodaphne perakensis* and *A. corneri* [25,26], and *N*-allyllaurolitsine from *Litsea lancifolia *[27]. These vasorelaxant effects may be mediated through the increased release of NO from endothelial cells. The mode of action of these indole alkaloids **1**, **2**, **4** and **5** on vasorelaxant activity is under investigation.

## 3. Experimental

### 3.1. General Procedures

Spectra were recorded on the following instruments: UV, Shimadzu UV-250 UV-visible spectrophotometer; IR, Perkin Elmer 1600; NMR, JEOL ECA 400 MHz; LCMS-IT-TOF, Shimadzu. All solvents, except those used for bulk extraction are AR grade. Silica gel 60 F_254_ for thin layer chromatography (TLC) was used for column chromatography. Glass and aluminum supported silica gel 60 F_254_ plates were used for TLC. TLC spots were visualized under UV light (254 and 365 nm) followed by spraying with Dragendorff’s reagent for alkaloid detection.

### 3.2. Plant Material

The bark of *Nauclea officinalis *was collected at Hutan Simpan Madek, Keluang, Johor, Malaysia by the phytochemical group of the Department of Chemistry, Faculty of Science, University of Malaya. The voucher specimen (KL 5655) of this plant has been deposited at the Herbarium of the Department of Chemistry, University of Malaya, Kuala Lumpur, Malaysia.

### 3.3. Extraction and Isolation

Dried, grounded bark of the plant (2.0 kg) was first defatted with hexane (17 litres) for one night. The dried materials then were extracted using CH_2_Cl_2_ (17 litres) twice for a 3-day period. The supernatant obtained was concentrated using rotary evaporator under reduced pressure to a volume of 500 mL and examined for its alkaloid content (using TLC and confirmed by spraying with Dragendorff’s reagent). The extract was finally concentrated to give crude alkaloids (11.0 g). The crude alkaloid (8.0 g) was subjected to column chromatography over silica gel using dichloromethane and methanol solvent (100:0, 99:1, 98:2, 97:3, 96:4, 95:5, 94:6, 90:10, 83:17, and 75:25) and finally with 100% methanol was used as eluent to obtain twenty fractions. Further purification of fraction 14 by Preparative Thin Layer Chromatography (PTLC) yielded alkaloid **1** (7.9 mg, MeOH-CH_2_Cl_2_; 97:3: saturated with NH_4_OH). Both known compounds **3** (1.5 mg, MeOH-CH_2_Cl_2_; 98:2: saturated with NH_4_OH) and **4** (7.2 mg, MeOH-CH_2_Cl_2_; 98:2: saturated with NH_4_OH) were obtained after purification of fraction 12, while the compounds **2** (8.5 mg, MeOH-CH_2_Cl_2_; 98:2: saturated with NH_4_OH) and **5** (10.1 mg, MeOH-CH_2_Cl_2_; 99:1: saturated with NH_4_OH) were obtained from fractions 7 and 6, respectively.

#### Naucline (**1**)

Brown amorphous solid, LCMS-IT-TOF at *m/z* 319.1450 ([M+H]^+^ for C_20_H_18_N_2_O_2_; UV (MeOH) 374, 215 nm; IR (CHCl_3_) λ_max_: 3188, 2924, 2859, 1639, 1572, 1533, 1496, and 1456 cm^−^^1^; ^1^H- and ^13^C-NMR: see Table 1.

### 3.4. Vasodilation Assay

A male Wistar rat weighing 260 g was sacrificed by bleeding from carotid arteries under anesthetization. A section of the thoracic aorta between the aortic arch and the diaphragm was removed and placed in oxygenated, modified Krebs-Henseleit solution (KHS: 118.0 mM NaCl, 4.7 mM KCl, 25.0 mM NaHCO_3_, 1.8 mM CaCl_2_, 1.2 mM NaH_2_PO_4_, 1.2 mM MgSO_4_, and 11.0 mMglucose). The aorta was cleaned of loosely adhering fat and connective tissue and cut into ring preparations 3 mm in length. The tissue was placed in a well-oxygenated (95% O_2_, 5% CO_2_) bath of 5 mL KHS solution at 37 °C with one end connected to a tissue holder and the other to a force-displacement transducer (Nihon Kohden, TB-611T). The tissue was equilibrated for 60 min under a resting tension of 1.0 g. During this time the KHS in the tissue bath was replaced every 20 min.

After equilibration, each aortic ring was contracted by treatment with 3 × 10^−7^ M PE. The presence of functional endothelial cells was confirmed by demonstrating relaxation to 1 × 10^−5^ M acetylcholine (ACh), and aortic ring in which 80% relaxation occurred, were regarded as tissues with endothelium. When the PE-induced contraction reached a plateau, each sample (**1**, **2**, **4** and **5**, 1 × 10^−5^ M) was added.

These animal experimental studies were conducted in accordance with the Guiding Principles for the Care and Use of Laboratory Animals, Hoshi University and under the supervision of the Committee on Animal Research of Hoshi University, which is accredited by the Ministry of Education, Science, Sports Culture, and Technology of Japan.

## 4. Conclusions

**Scheme 1:**
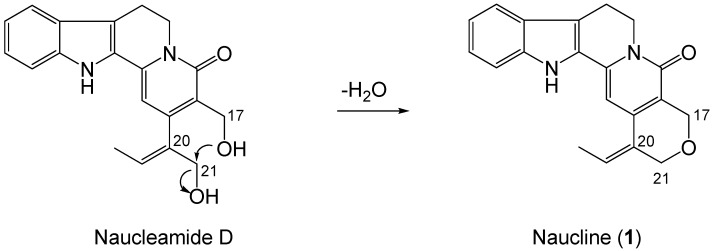
A proposed biogenesis process of naucleamide D to naucline (**1**).

In conclusion, five alkaloids were isolated from the bark of *Nauclea officinalis* of which one is a new pyranoindoloquinolizidinone alkaloid, which has been named naucline (**1**). The formation of **1** was proposed to ocurr as shown in Scheme 1. The biogenetic precursor of **1** would be naucleamide D which could undergo a dehydration involving the hydroxyl group at C-21 to form naucline (**1**). Naucline (**1**) showed a moderate vasorelaxant activity (90% relaxation at 1 × 10^−5^ M) whereas angustine (**2**), nauclefine (**4**) and naucletine (**5**) showed excellent vasorelaxant activity (more than 90% relaxation at 1 × 10^−5^ M) on an isolated rat aorta.
